# *Garcinia dulcis* Flower Extract Alters Gut Microbiota and Fecal Metabolomic Profiles of 2K1C Hypertensive Rats

**DOI:** 10.3390/nu15020268

**Published:** 2023-01-05

**Authors:** Phornphan Sornchuer, Nattaya Thongsepee, Lampet Wongsaroj, Kritsakorn Saninjuk, Suphot Wattanaphansak, Pornkanok Pongpamorn, Atchara Paemanee, Pongsakorn Martviset, Pathanin Chantree, Kant Sangpairoj

**Affiliations:** 1 Department of Preclinical Science, Faculty of Medicine, Thammasat University, Khlong Luang 12120, Thailand; 2 Thammasat University Research Unit in Nutraceuticals and Food Safety, Faculty of Medicine, Thammasat University, Khlong Luang 12120, Thailand; 3 Porcinotec Co., Ltd., Nonthaburi 11000, Thailand; 4 Departments of Veterinary Medicine, Faculty of Veterinary Science, Chulalongkorn University, Bangkok 10330, Thailand; 5 National Omics Center, National Science and Technology Development Agency (NSTDA), Khlong Luang 12120, Thailand

**Keywords:** gut microbiota, metabolites, 2K1C rat, *Garcinia dulcis*, hypertension

## Abstract

*Garcinia dulcis* (GD) extract has been found to have anti-hypertensive properties in animal studies. GD can also alter the colonic microbiota of rats. However, the effects of GD on changes in the gut microbiota and metabolomic profiles of normotensive and hypertensive rats are currently unknown. The purpose of this study was to evaluate changes in the gut microbiota and metabolomic profiles of 2-kidneys-1 clip (2K1C) hypertensive rats after feeding with GD flower extract. Rats were randomly divided into the following 4 groups: sham operation (SO) receiving corn oil (CO) (SO + CO), SO receiving GD (SO + GD), 2K1C receiving corn oil (2K1C + CO) and 2K1C receiving GD (2K1C + GD). Body weight (BW) and systolic blood pressure (SBP) were measured weekly throughout the study. Gut microbiota and fecal metabolites were measured from fresh fecal contents. Alpha diversity results demonstrated a similar microbial richness and diversity between groups. Linear discriminant analysis (LDA) effect size (LEfSe) suggested that GD treatment affected gut microbial community structure in both hypertensive and normotensive rats. Feeding rats with GD caused metabolic alterations that rendered 2K1C + GD rats similar to SO + CO and SO + GD rats. Findings suggest that the impact of GD on gut microbiota and metabolite profiles may be related to its anti-hypertensive properties.

## 1. Introduction

*Garcinia dulcis* (GD), which belongs to the *Guttiferae* family, grows mainly in the American tropical and Southeast Asia regions, including southern Thailand [1]. The extracts of various parts of GD have been studied both in vitro and in vivo [2]. The major bioactive compounds of GD fruit are morelloflavone and garcinol [3], which have hepatoprotective and anti-proliferative properties [4,5]. The hexane-insoluble fraction of the GD flower extract, which is mainly composed of camboginol and morelloflavone, can lower the arterial blood pressure of 2-kidney-1-clip (2K1C) renovascular hypertensive rats [6]. Camboginol and morelloflavone have antioxidant properties as effective scavengers of the DPPH (2,2-diphenyl-l-picrylhydrazyl) radical and the nitric oxide radical [3,5]. Since these compounds are efficient antioxidants, their protective effects on the cardiovascular system are clear.

Hypertension is an important risk factor for atherosclerotic cardiovascular disease. Atherosclerotic disease can be triggered by genetic, lifestyle, environmental and hormonal factors as well as inflammatory and hemodynamic changes [7]. Renovascular hypertension (RVH) is a secondary form of hypertension that is triggered by atherosclerotic disease or fibromuscular dysplasia (FMD) of the renal arteries [8]. The most prominent causes of RVH are the activation of the renin-angiotensin system (RAS) as well as the presence of inflammatory and oxidative stress responses [9,10]. Animal models of 2K1C have shown that nerves from the ischemic kidney partially contribute to the development of hypertension and renal alterations, such as proteinuria and intrarenal RAS activation [11]. The 2K1C model is therefore widely used to study RVH [12].

It has previously been reported that the gut microbiota plays a crucial role in the development and pathogenesis of hypertension [13,14]. Studies involving hypertensive rat models and human hypertensive patients suggest that gut microbial dysbiosis, defined as decreased microbial richness and diversity and an increased *Firmicutes*/*Bacteroidetes* ratio, is closely related to hypertension [15]. There was a significant difference in the gut microbial community structure of 2K1C rats compared with the sham operation (SO) group, notably reduced abundance of *Firmicutes*, increased *Bacteroidetes*, increased relative abundance of *Escherichia coli* and reduced relative abundance of short chain fatty acid (SCFA)-producing strains [16]. RVH therefore impacts gut microbial community structure in a rat model. Moreover, SCFAs produced by gut microbiota induced an increased nitric oxide (NO) level in an AMPK (adenosine monophosphate-activated protein kinase)-dependent manner [17]. NO plays a major role in the regulation of arterial blood pressure (ABP), and reduced NO bioavailability is present in the setting of arterial hypertension [18].

Increased consumption of fruit and vegetables may prevent chronic diseases, including cardiovascular disease [19]. Dietary interventions may correct gut microbiota and have been proposed as an innovative nutritional therapeutic strategy for hypertension. A GD flower extract has been shown to have anti-hypertensive properties in a rat model. However, the effects of GD flower extracts on gut microbiota and metabolomic alterations in 2K1C rats have not been reported. A focused study of the biological effects of GD flower extract on the gut microbiota and metabolomic profiles might provide information on the potential pharmaceutical uses of this plant.

## 2. Materials and Methods

### 2.1. Animals

Twenty-four 5-week-old male Wistar rats were purchased from Siam Nomura International Co., Ltd. (Bangkok, Thailand). The animals were housed (2 rats/cage) at the Animal Laboratory Center of Thammasat University and maintained under standard conditions, which included room temperature of 22 ± 1 °C, relative humidity of 30–70% RH, light intensity of 130–325 Lux and 12/12 h dark-light cycle and were fed with a commercial pellet food (CE-2) and reverse osmosis water *ad libitum*. The diet CE-2 consists of 8.9% moisture, 24.9% crude protein, 4.6% crude fat, 4.1% crude fiber, 6.6% crude ash and 51.0% nitrogen-free extract, with 344.9 Kcal/100 g of total energy. Rats were acclimatized for a week before experimentation. All experimental protocols adhered to the NIH Guiding Principles in the Care and Use of Animals and were approved by the Thammasat University Animal Care and Use Committee Protocol No. 025/2022.

### 2.2. Induction of Hypertensive Model

After acclimatization, rats were randomly divided into two groups (12 animals each), for either 2K1C or SO, to develop a hypertensive or normotensive rat model, respectively. The operative procedure was performed as previously described [6]. In brief, rats were anaesthetized via inhalation of isoflurane according to a standard protocol. Surgery was performed to place a silver clip around the left renal artery. Rats were retained for 4 weeks after surgery to permit the development of hypertension.

### 2.3. Treatment of GD Flower Extract

**Figure 1:**
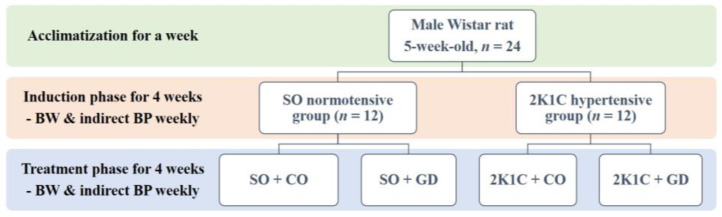
Schematic of the experimental design for this study.

GD flowers were collected from Songkhla province, Thailand. GD extraction and purification procedures were previously described by Thongsepee et al., 2020 [20]. The major chemical components of the GD flower extract were morelloflavone and camboginol. The extract was dissolved in a small amount of dimethyl sulfoxide (0.3% DMSO; Sigma-Aldrich, Darmstadt, Germany), then further dissolved in corn oil (Mazola, Bangkok, Thailand). The GD flower extract was administered via oral gavage at a dose of 50 mg/kg BW daily for 4 weeks after hypertension was induced. The control groups received corn oil (CO) at the same dose (2.5 mL/kg BW). A schematic of the experimental design of this study is shown in Figure 1. Rats were thus divided into four sub-groups (*n* = 6/group): (I) SO + CO; (II) SO + GD; (III) 2K1C + CO; and (IV) 2K1C + GD.

### 2.4. Indirect BP and BW Measurement

In order to monitor the development of hypertension, systolic blood pressure (SBP) was measured using the BP tail cuff (non-invasive BP) and the PowerLab system (model 26T, ADInstruments, Bella Vista, NSW, Australia) before and weekly following the inductive surgery. Body weight (BW) was recorded weekly throughout the study. All measurements were performed in triplicate, and the average values were obtained. Changes in SBP (∆SBP) and BW (∆BW) during both the inductive and treatment phases were reported.

### 2.5. Stool Sample Collection and DNA Extraction

Fresh fecal content was collected sterilely from the rat’s cecum after sacrifice and stored at −80 °C. Metagenomic DNA for prokaryotes was isolated using the QIAamp PowerFecal Pro DNA Kit (Qiagen, Hilden, Germany) according to the manufacturer’s protocols. Briefly, 0.25 g of fecal samples were extracted. The quality of the extracted DNA was measured using the DeNovix Fluorometer (Wilmington, DE, USA).

### 2.6. Metagenomic Sequencing and Bioinformatic Analysis

The prokaryotic 16S rRNA gene at the V3–V4 region was performed using the Qiagen QIAseq 16S/ITS Region panel (Qiagen, Hilden, Germany). 16S rRNA amplicons were labeled with different sequencing adaptors using the QIAseq 16S/ITS Region Panel Sample Index PCR Reaction (Qiagen, Hilden, Germany). The quality and quantity of approximately 630 bp DNA libraries were evaluated using QIAxcel Advanced (Qiagen, Hilden, Germany) and the DeNovix QFX Fluorometer, respectively. The 16S rRNA libraries were sequenced using an Illumina Miseq600 platform (Illumina, San Diego, CA, USA).

Raw sequences were categorized into groups based on 5′ barcode sequences. The sequences were processed using the DADA2 v1.16.0 pipeline (https://benjjneb.github.io/dada2/ accessed on 5 July 2022). The DADA2 pipeline describes microbial diversity and community structures using unique amplicon sequence variants (ASVs) [21]. Microbial taxa were classified using Silva version 138 as the reference database [22]. The Alpha diversity index (Chao1 richness, Shannon and PD whole tree) was computed using DADA2 software. For beta diversity, non-metric multidimensional scaling (NMDS) based on Bray–Curtis dissimilarity and principal coordinate analysis (PCoA) on unweighted Unifrac were plotted using Phyloseq data [23]. Linear discriminant analysis effect size (LEfSe) was used to identify bacterial biomarkers. Pairwise comparison of alpha diversity (observed ASVs, Chao1, Shannon and PD whole tree) was calculated using the Kruskal–Wallis test (*p* < 0.05). Permutational multivariate analysis of variance (PERMANOVA) was performed to identify significant differences in beta diversity at *p* < 0.05. The Kruskal–Wallis sum-rank test was also used in LEfSe analysis to identify bacterial biomarkers that differed significantly in abundant taxon between sample groups. Bioinformatics analyses of the microbial data were performed by Porcinotec Co., Ltd. (Nonthaburi, Thailand).

### 2.7. Fecal Metabolite Analysis

For fecal extraction, 100 mg of each fecal sample was mixed with 1 mL of 50% acetonitrile and vortexed for 5 min. The sample was centrifuged at 3220 RCF at 10 °C for 10 min, after which 20 µL of the supernatant was transferred to a microcentrifuge tube for derivatization. A volume of 10 µL of 200 mM 3-NPH·HCl in 50% acetonitrile was added to the sample, followed by 10 µL of 120 mM EDC·HCl in 50% acetonitrile + 6% pyridine. The reaction was carried out using an Eppendorf ThermoMixer at 40 °C for 30 min, shaking at 1100 rpm. The mixture was cooled down on ice for 5 min and diluted to 1 mL with 10% acetonitrile. This solution was then filtered through a 0.22 µm nylon membrane filter and analyzed by UHPLC-Q-Orbitrap HRMS. Relative quantification of the fecal metabolites was performed with a Vanquish Horizon UHPLC (ultra-high performance liquid chromatography) system coupled to a Q Exactive HF-X Hybrid Quadrupole-Orbitrap mass spectrometer (Thermo Fisher Scientific, San Jose, CA, USA). Reversed-phase chromatography was performed using an Xselect HSS T3 column (2.1 × 100 mm, 2.5 μm, Waters Corp., Milford, MA, USA) with a VanGuard precolumn (2.1 × 5 mm, Waters Corp., Milford, MA, USA). The mobile phases were water + 0.1% formic acid (A) and acetonitrile + 0.1% formic acid (B). Gradient elution was performed at 0.35 mL/min: 0–2 min 15% B, 2–11 min 15–55% B, 11–12 min 55–100% B, 12–13 min 100% B, 13–13.5 min 100–15% B and 13.5–18.5 min 15% B. The column temperature was maintained at 40 °C, and the injection volume was 2 µL.

MS detection was performed using an electrospray ionization source in the negative mode at the following settings: capillary temperature = 275 °C, auxiliary gas temperature = 400 °C, sheath gas flow rate = 45 arb, auxiliary gas flow rate = 10 arb, sweep gas flow rate = 2 arb, funnel RF level = 40 arb, spray voltage = 2.5 kV and collision energy = 30 arb. Metabolites were monitored in a data-dependent mode using a full scan resolution = 120,000 FWHM at *m*/*z* 50–500 and a MS2 resolution = 30,000 FWHM. Standard compounds, including acetic acid, propionic acid, butyric acid, isobutyric acid, 2-methylbutyric acid, valeric acid, isovaleric acid, malonic acid, succinic acid and l-phenylalanine, were prepared in 50% acetonitrile at 1000 ppm and derivatized in the same way as the experimental samples. Their MS spectra were stored within the mzVault 2.3. (Thermo Fisher Scientific, USA) in-house library. All raw files were processed and analyzed with Compound Discoverer 3.3 (Thermo Fisher Scientific, USA). Pooled samples of QCs were used for data normalization. Fecal metabolites were measured at the National Omics Center, NSTDA (Pathum Thani, Thailand). The population distribution of all fecal samples was assessed using principal-component analysis (PCA). Hierarchical cluster analysis was performed using MetaboAnalyst [24] and presented as dendrograms on the heatmap.

### 2.8. Statistical Analysis

Rat body weight and BP data were reported as mean ± standard error of the mean (S.E.M.). A one-way analysis of variance (ANOVA) test with Tukey’s pairwise comparisons test was performed using GraphPad Prism 9.0 software (LaJolla, CA, USA) to identify significance between the different groups. A *p* value < 0.05 was considered statistically significant.

## 3. Results

### 3.1. Effects of GD Extract on Changes in Body Weight and SBP Levels

**Figure 2:**
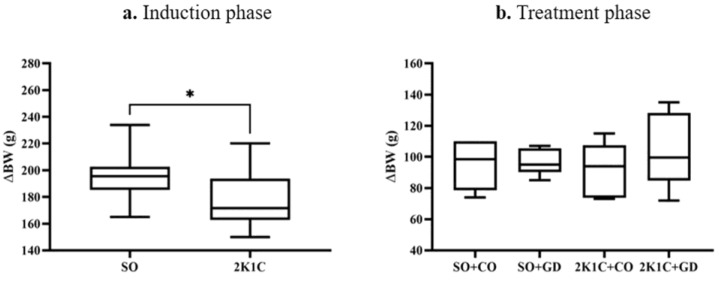
Body weight changes (∆BW, g) in the sham-operative (SO) and 2K1C groups during the hypertension induction phase (**a**) and during the treatment phase with GD flower extract (**b**). Data are expressed as mean ± S.E.M. * *p* < 0.05 between groups.

During the hypertension induction phase, the body weight changes (ΔBW) of the 2K1C rats were significantly lower than those of the SO rats (SO: 196 ± 6 g, 2K1C: 177 ± 6 g, *p* < 0.05, Figure 2a). There were no significant differences in ΔBW during the treatment phase (SO + CO: 95 ± 6 g, SO + GD: 97 ± 3 g, 2K1C + CO: 93 ± 7 g, 2K1C + GD: 103 ± 10 g, Figure 2b). This finding suggests that induced hypertension can affect rat BW. However, BW ultimately recovers. Treatment with GD flower extract had no effect on BW.

**Figure 3:**
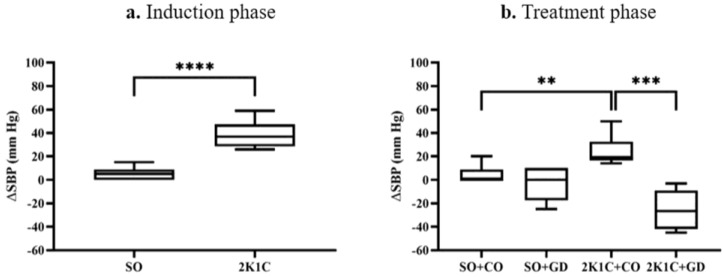
Changes in the tail-cuff BP (∆SBP, mm Hg) of the sham-operative (SO) and 2K1C groups during the hypertension induction phase (**a**) and the GD flower extract treatment phase (**b**). Data are expressed as mean ± S.E.M. ** *p* < 0.01, *** *p* < 0.001, **** *p* < 0.0001 between groups.

During the hypertension induction phase, the changes in the tail-cuff BP (∆SBP) of the 2K1C group were significantly higher than those of the SO group (2K1C: 38.50 ± 3.01, SO: 4.58 ± 1.44 mm Hg, *p* < 0.0001). The ∆SBP in the 2K1C + CO group during the treatment phase was still significantly higher than the SO + CO group (2K1C: 24.50 ± 5.41, SO: 4.17 ± 3.27, *p* < 0.01). The ∆SBP in the 2K1C + GD group (−25.50 ± 6.74 mm Hg) was significantly decreased compared with the 2K1C + CO group (*p* < 0.001). There was no difference in ∆SBP between the SO + GD (−3.33 ± 5.73 mm Hg) and SO + CO groups (Figure 3). This finding suggests that hypertension was successfully induced and that GD flower extract possesses anti-hypertensive properties.

### 3.2. Effects of GD Extract on Alteration of Gut Microbiota

A 16S rRNA gene-based analysis was used to assess bacterial communities in the rat fecal samples. Data was obtained for six replicates from each group. High-quality reads of 16S rRNA after processing were 1,313,567 reads. The 44,729 sequencing depths allowed data normalization for analysis of taxonomic compositions at the genus level (Table 1).

**Figure 4:**
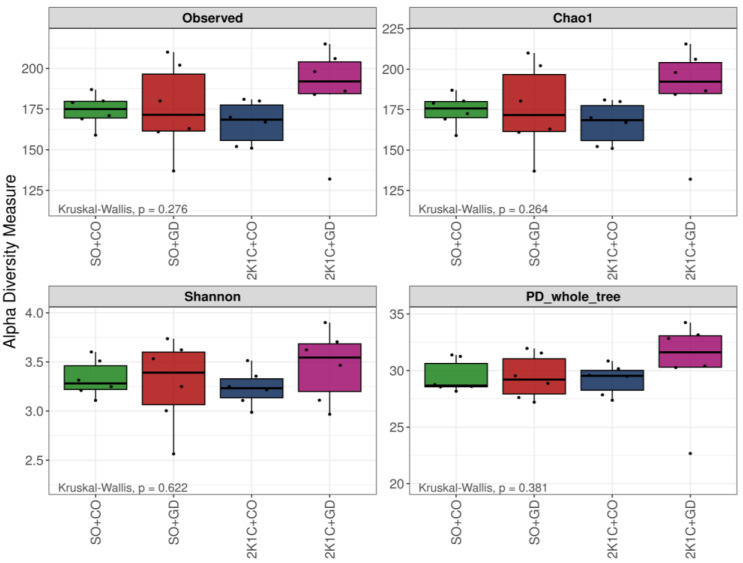
Box plots of the alpha diversity (observed number of ASVs, Chao1, Shannon and the PD whole tree) of each sample group. Black dots represent the individual samples in each group. SO, sham operation; CO, corn oil; 2K1C, 2-kidneys-1 clip; GD, *Garcinia dulcis* flower extract.

**Figure 5:**
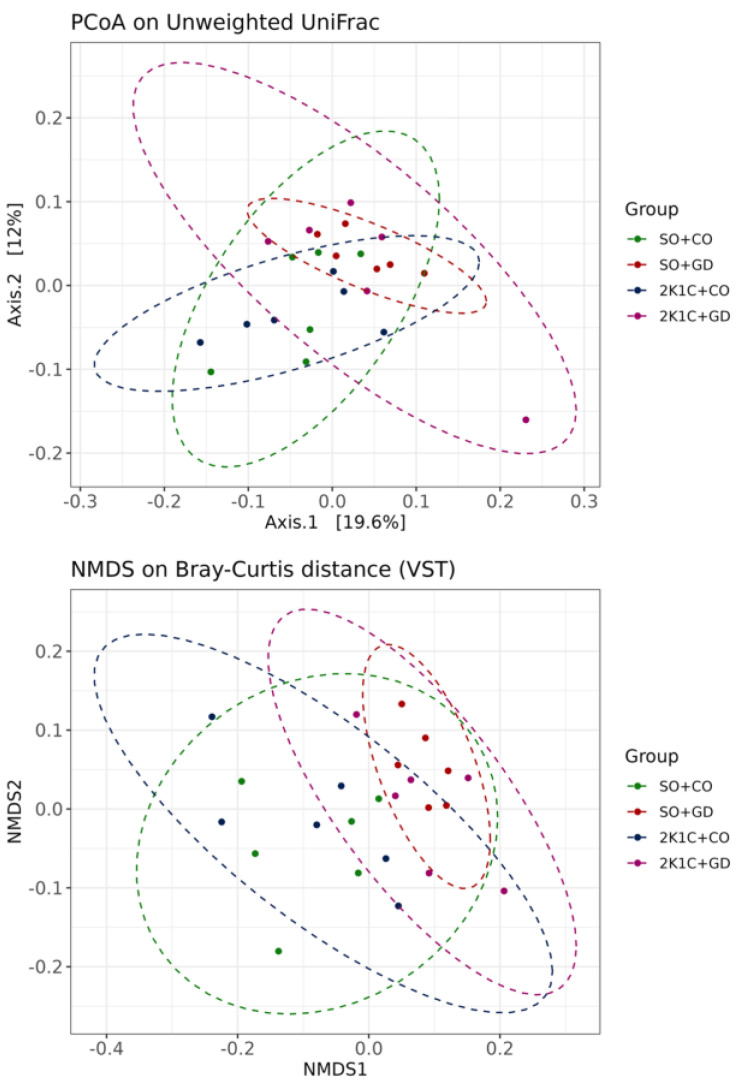
Beta-diversity plots. Two-dimensional PCoA on unweighted Unifrac distances and NMDS based on Bray–Curtis dissimilarity were generated.

Alpha diversity revealed that there was no significant difference in ASV abundance, bacterial richness (Chao1), diversity (Shannon) and phylogenetic diversity (PD) in the whole tree between the different groups (*p* > 0.05) (Figure 4). The overall beta-diversity of the gut microbiota, in terms of unweighted UniFrac distance and nonmetric multidimensional scaling (NMDS), was analyzed (Figure 5). The unweighted UniFrac principal-coordinate analysis (PCoA) and NMDS based on Bray–Curtis distance showed that the microbiota communities were clearly distinct within each group (PERMANOVA test; *p* = 0.01 and *p* = 0.001, respectively). Pairwise testing between each sample group using unweighted UniFrac PCoA found statistically significant differences in 2K1C + CO vs. SO + GD and SO + CO vs. SO + GD (*p* < 0.05). Moreover, pairwise analysis of NMDS based on Bray–Curtis distance showed that 2K1C + CO was significantly different from 2K1C + GD and SO + GD (*p* = 0.03 and *p* = 0.01, respectively), and SO + CO was significantly different from 2K1C + GD and SO + GD (*p* = 0.006). This finding suggests that both hypertension induction and GD flower extract treatment affected the gut microbial community.

### 3.3. Taxonomic Structure of the Bacterial Communities

**Figure 6:**
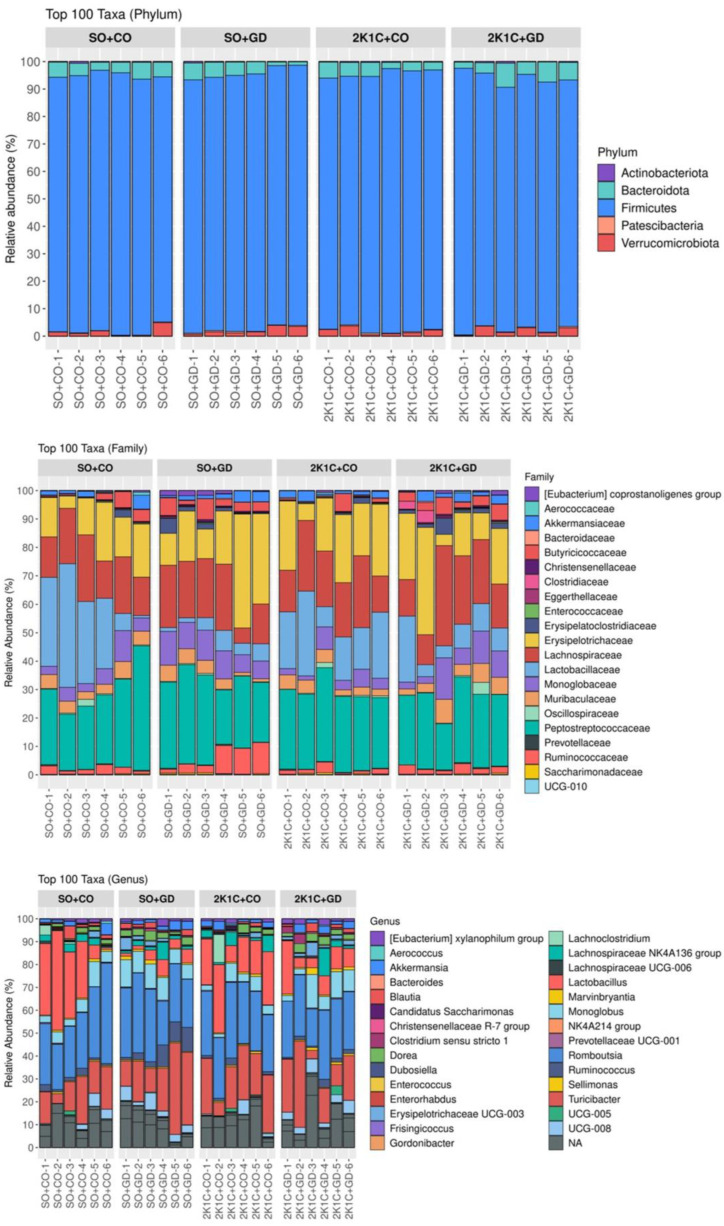
Taxonomic profiles of the bacterial communities illustrated at the phylum, family and genus levels. SO, sham operation; CO, corn oil; 2K1C, 2-kidneys-1 clip; GD, *Garcinia dulcis* flower extract.

The microbiota compositions of the different taxa profiles (phylum, family and genus) were depicted as bar charts (Figure 6). A total of eight different bacterial phyla were identified. Only five enriched phyla were shown for the top 100 taxonomic classifications. The bacteria in the phylum *Firmicutes* were highly prevalent (avg. 91.67 ± 18.71%), followed by *Bacteroidota* (avg. 5.41 ± 1.1%) and *Verrucomicrobiota* (avg. 1.82 ± 0.37%). Analysis of bacterial community structure at the family level showed that *Peptostreptococcaceae*, *Lachnospiraceae*, *Erysipelotrichaceae*, *Lactobacillaceae*, *Muribaculaceae*, *Monoglobaceae*, *Ruminococcaceae* and *Akkermansiaceae* were the most dominant in the fecal samples. *Romboutsia*, *Turicibacter*, *Lactobacillus*, the *Lachnospiraceae* NK4A136 group, *Akkermansia*, *Monoglobus* and *Ruminococcus* were the major genera identified at the genus level.

**Figure 7:**
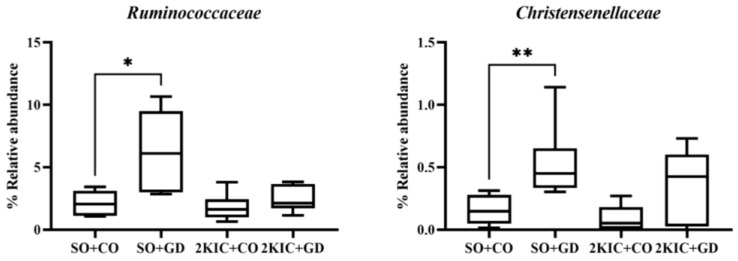
Composition alterations in gut microbiota at the family level. SO, sham operation; CO, corn oil; 2K, 2-kidneys-1 clip (2K1C); GD, *Garcinia dulcis* flower extract. * *p* < 0.05 and ** *p* < 0.01.

Changes in the composition of the gut microbiota at different taxonomic levels were observed. There were no statistically significant phylum-level differences between the groups. At the family level (Figure 7), an increased relative abundance of Ruminococcaceae and Christensenellaceae was observed in SO + GD compared with SO + CO. However, there were no differences in bacterial families in SO + CO vs. 2K1C + CO or 2K1C + CO vs. 2K1C + GD.

**Figure 8:**
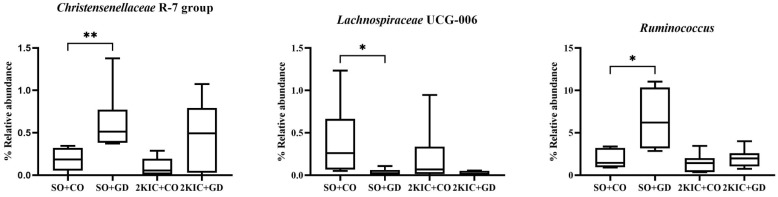
Alterations in gut microbiota composition at the genus level. SO, sham operation; CO, corn oil; 2K1C, 2-kidneys-1 clip; GD, *Garcinia dulcis* flower extract. * *p* < 0.05 and ** *p* < 0.01.

At the genus level (Figure 8), the relative bacterial abundance of members of the *Christensenellaceae* R-7 group and *Ruminococcus* were significantly increased in SO + GD compared with the SO + CO group, while that of *Lachnospiraceae* UCG-006 was significantly decreased. However, treatment with GD flower extract did not alter the relative abundance of these three bacteria in the 2K1C group. Moreover, the hypertensive conditions (2K1C + CO) created in this study did not affect the abundance of these three bacteria compared with the SO + CO control group.

**Figure 9:**
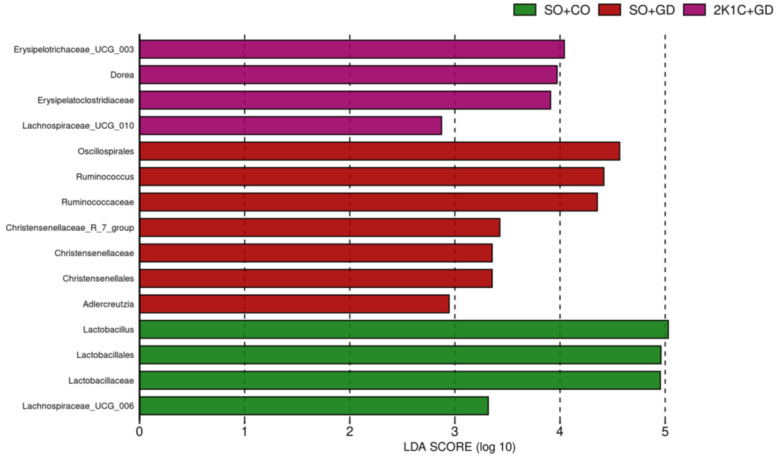
Linear discriminant analysis (LDA) effect size (LEfSe) bar plot representing the significant taxon of each group. The length of the bar represents a log10 transformed LDA score. The colors represent which group that taxon is more prevalent in compared with other groups. SO, sham operation; CO, corn oil; 2K1C, 2-kidneys-1 clip; GD, *Garcinia dulcis* flower extract.

Linear discriminant analysis (LDA) effect size (LEfSe) was used to identify significantly higher taxonomy or bacterial biomarkers that can explain differences in taxa between groups (Figure 9). Bacterial taxa with LDA scores greater than 2 were used to identify important taxonomic differences between groups. Bacteria in *Lactobacillaceae*, *Lactobacillales*, *Lactobacillus* and *Lachnospiraceae_UCG_006* were the core gut microbiota in the SO + CO group (*p* < 0.05). Members of *Oscillospirales*, *Ruminococcaceae*, *Ruminococcus*, *Christensenellales*, *Christensenellaceae*, *Christensenellaceae_R_7_group* and *Adlercreutzia* contributed to the dominant biomarkers in SO + GD (*p* < 0.05). *Erysipelotrichaceae_UCG_003*, *Dorea*, *Erysipelatoclostridiaceae* and *Lachnospiraceae_UCG_010* were highly prevalent in the 2K + GD group (*p* < 0.05). This finding suggests that GD flower extract treatment affected the gut microbial community structure in both normotensive and hypertensive rats.

### 3.4. Effects of GD Extract on Fecal Metabolite Profiles

**Figure 10:**
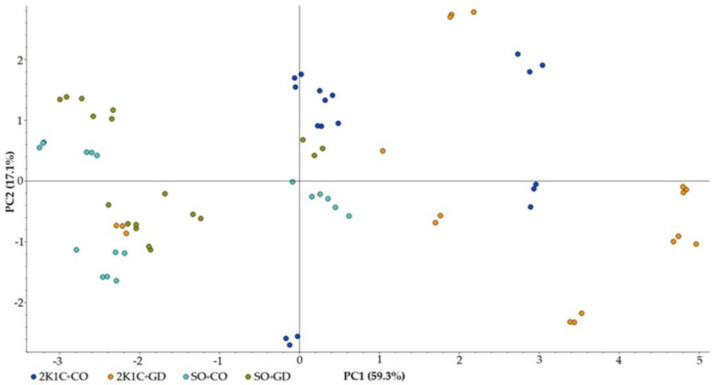
Scores plot of the principal-component analysis (PCA) showing correlations between the rat groups. Injections were conducted in triplicate per rat (*n* = 6 rats per group).

To assess changes in metabolomic profiles in response to changes in gut microbiota induced by each treatment condition, metabolomic profiling was performed using LC-MS. The identified fecal metabolites were classified as SCFAs (acetic acid, propionic acid, butyric acid, valeric acid, 2-methylbutyric acid, isobutyric acid and isovaleric acid), organic acids (malonic acid and succinic acid) and amino acids (l-phenylalanine). Principal-component analysis (PCA) showed that the SO + CO and SO + GD rats had a similar variance for PC1, while the 2K1C + CO and 2K1C + GD rats had a variance in the other direction (Figure 10), which indicated that there were differences in the levels of metabolites between normotensive and hypertensive rats. Almost all of the 2K1C + GD rats (5/6) were positioned on the same side of PC2 as the SO + CO (4/6) and SO + GD (3/6) rats, suggesting that feeding hypertensive rats with GD altered their metabolite profiles to have similar patterns to normotensive rats.

**Figure 11:**
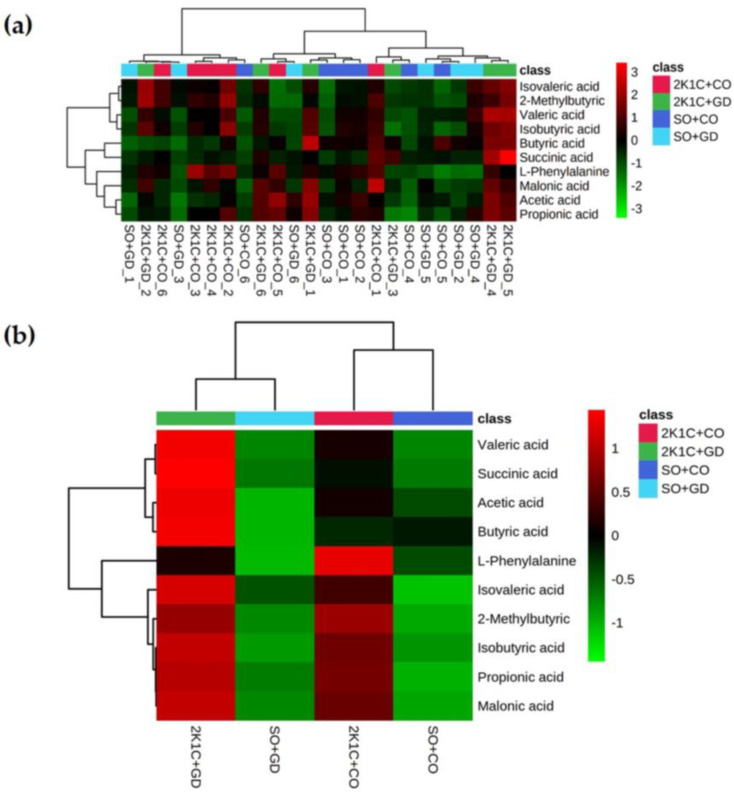
Hierarchical clustering heatmap between the metabolites and rats using Pearson distance measure and the Ward cluster algorithm; (**a**) all 12 rats and (**b**) averages of each rat group. The dendrogram on the left represents dissimilarities between the metabolite clusters, and the dendrogram above the heat map represents similarities between the rats.

Hierarchical cluster analysis on the heatmap (Figure 11a) showed that the rats were separated into two major groups. 2K1C + CO rats were clustered under a different branch from 2K1C + GD, SO + CO and SO + GD rats, which indicated that feeding GD caused metabolic alterations that render 2K1C + GD rats to be more similar to SO + CO and SO + GD rats than 2K1C + CO rats (Figure 10a). The hypertensive rats (2K1C) showed higher fecal SCFA concentrations compared to normotensive rats (Figure 11b). 2K1C + CO rats showed upregulated levels of l-phenylalanine, isovaleric acid, 2-methylbutyric acid, isobutyric acid, propionic acid and malonic acid and downregulated levels of valeric acid, succinic acid, acetic acid and butyric acid. However, feeding 2K1C rats with GD allowed the levels of valeric acid, succinic acid, acetic acid and butyric acid to be restored, as observed in SO rats, where all metabolites were detected at similar levels (Figure 11b).

## 4. Discussion

Various components of *Garcinia dulcis* (GD) contain an abundance of bioactive compounds with anti-atherosclerosis, anti-bacterial, anti-cancer and anti-hypertensive properties [3]. Studied in rats with diet-induced metabolic syndrome, treatment with GD fruit rind powder that was enriched with morelloflavone and garcinol inhibited inflammatory processes, suppressed appetite, increased fat metabolism and altered the colonic microbiota [2]. The hexane-insoluble fraction of the GD flower extract composed of 2 bioactive compounds, morelloflavone and garcinol (aka. Camboginol), exhibited anti-hypertensive and diuretic properties in a 2K1C RVH model [6]. Activation of RAS in the 2K1C RVH model contributed to changes in water and electrolyte balance and upregulated the sympathetic nervous system, which induced an elevated BP. Several studies have indicated that some bacterial taxa were different between the hypertensive and normotensive groups in both animal models and human studies. Hypertension has been associated with gut dysbiosis, characterized by reduced microbial abundance, richness and diversity [25]. The involvement of the gut microbiota in the regulation of hypertension in 2K1C models has been previously established [17]. Our findings agree with previous studies [6] that BPs were lower in the 2K1C hypertensive group but not the normotensive group after treatment with GD extract. The proposed anti-hypertensive effects of GD extract include anti-inflammatory, NO-dependent vasorelaxation and diuresis [6]. We therefore sought to determine if these conditions could affect the gut microbiota. Moreover, we were also interested in the effects of GD flower extract on the gut microbiota and fecal metabolomic profiles in 2K1C hypertensive rats.

We found that gut microbial communities varied between experimental groups. LEfSe analysis indicated that *Lactobacillaceae*, *Lactobacillales*, *Lactobacillus* and *Lachnospiraceae*_UCG_006 were the core gut microbiota in the normotensive group, SO + CO. *Lactobacilli* is associated with protection against infection, enhanced recovery after enteric infections, reduced colitis pathology and improved cognitive function [26]. Several studies indicated that *Lactobacilli* is known to have BP-lowering effects [7,27,28]. Members of *Oscillospirales*, *Ruminococcaceae*, *Ruminococcus*, *Christensenellales*, *Christensenellaceae*, *Christensenellaceae*_R_7_group and *Adlercreutzia* were dominant in SO + GD, while *Erysipelatoclostridiaceae*, *Erysipelotrichaceae*_UCG_003, *Lachnospiraceae*_UCG_010 and *Dorea* were highly prevalent in the 2K + GD group. The genus *Erysipelotrichaceae*_UCG_003 of family *Erysipelatoclostridiaceae* has been linked with the formation of isobutyrate, isovalerate and valerate [29]. *Lachnospiraceae* are known for including many species able to produce SCFAs, which have often been revealed to have a positive association with health [30]. The *Lachnospiraceae* NK4A136 group has been positively correlated with the production of both acetic acid [30] and butyric acid [31,32]. *Dorea* has been reported to produce acetate and lactate, which may serve as substrates for butyrate production [33]. Nevertheless, *Dorea* has either pro or anti-inflammatory roles depending on the neighboring gut bacteria and/or existing nutrients [34].

SCFAs, free fatty acids containing fewer than six carbons, include acetic acid, propionic acid, butyric acid and valeric acid. SCFAs, especially acetate, propionate and butyrate, are considered the most common end products of microbial fermentation. They function as energy resources for the gut epithelium and play a role in the host immune system. The hypertensive rats (2K1C) showed higher fecal SCFA concentrations compared to normotensive rats, which was similar with previous findings in human fecal samples [35]. Hypertensive subjects exhibited significantly higher levels of SCFAs in feces while presenting depleted levels of plasma SCFAs. It is suggested that a lower efficiency in the absorption of SCFAs could occur in hypertensive subjects [35]. Similar results have been reported in animal studies [36,37]. In this study, feeding hypertensive rats with GD altered their SCFA levels to have a similar pattern to normotensive rats. SCFAs have been previously reported to exert an antihypertensive effect [38]. SCFAs modulate blood pressure regulation through G-protein-coupled receptors, directly interrupting the intrarenal renin-angiotensin system [39]. Intramedullary infusion of sodium butyrate in uninephrectomized rats resulted in the reversal of angiotensin-II-induced glomerulosclerosis and decreased the expression of the (pro)renin receptor, angiotensinogen, renin, angiotensin-I-converting enzyme and renal inflammatory markers [40]. A high-fiber diet that promotes the growth of acetate-producing bacteria could reverse renal fibrosis in a deoxycorticosterone acetate hypertension model [36]. These findings suggest that SCFAs participate in the immune response and can also reverse kidney pathology [41]. Acetate and propionate produced by gut microbiota are also able to decrease systemic inflammation and artherosclerotic lesions [38]. Lactate and butyrate are linked to BP modulation through vasodilation and vasoconstriction [38]. Malonate can inhibit mitochondrial ROS production, which subsequently reduces AngII-induced O_2_^•−^ production in the cytoplasm [42]. Colon-derived valeric acid rapidly penetrated from the gastrointestinal tract to the eyes in a rat model, decreasing intraocular and arterial blood pressures significantly [43].

The increased levels of l-phenylalanine were observed in 2K1C hypertensive rats but declined after feeding with GD flower extract. Phenylalanine is an aromatic amino acid that cannot be synthesized in vivo [44]. Phenylalanine absorbed by the host is either utilized by the host or gut microbiota. The gut symbiont *Clostridium sporogenes* metabolizes phenylalanine into its corresponding propionic acid derivatives, phenylpropionic acid (PPA) and phenylacetic acid [45]. Hypertensive induction in this study might have triggered gut dysbiosis, which subsequently affected phenylalanine metabolism. The fecal phenylalanine in hypertensive rats would have therefore been upregulated. Treatment with GD flower extract may compensate for this event. However, the full effects of GD flower extract on phenylalanine metabolism require further characterization.

Overall, our study provides a better understanding of the biological effects of GD flower extract on the aspect of the gut–kidney axis. Limitations of this study include the lack of a serum profile of the fecal metabolites as well as hematological tests, which can provide data on the effects of gut microbiota and metabolite changes on the host. Furthermore, the dose and duration of GD treatment should be verified in future studies in order to gain insight into the mechanism of action of GD extract on the host. Further functional characterization both in vitro and in vivo will be necessary to fully characterize the role of bacterial species on the gut–kidney axis.

## 5. Conclusions

In conclusion, the effects of GD flower extract on the gut microbiota and fecal metabolites of 2K1C hypertensive rats were characterized. GD flower extract altered the core gut microbiota and fecal metabolite profiles of 2K1C hypertensive rats. Feeding hypertensive rats with GD flower extract could alter the SCFA levels (valeric acid, succinic acid, acetic acid and butyric acid) to have a similar pattern to normotensive rats. This finding is in agreement with the LEfSe analysis, which showed the predominant abundance of SCFA-producing bacteria (*Erysipelotrichaceae*, *Lachnospiraceae* and *Dorea*) in the fecal contents of 2K1C + GD rats. Therefore, further studies should be conducted to determine the serum profiles of GD flower extract treatment and the long-term effects of the extract on the gut microbiota in the host.

## Figures and Tables

**Table 1:** Estimated sequencing coverage and alpha diversity indices of the bacterial taxonomic profiles of the 16S rRNA gene sequences at the genus level.

Group	Sample ID	Raw Reads	Quality Reads	OTUs	Chao	Shannon	PD_Whole_Tree
SO + CO	SO + CO-1	59,079	50,854	171	171	3.320187	28.174087
	SO + CO-2	64,823	53,031	157	157.20	3.269554	28.537433
	SO + CO-3	62,658	52,104	181	181	3.593931	28.849749
	SO + CO-4	79,871	67,816	167	168	3.203351	28.555159
	SO + CO-5	57,408	48,821	187	187	3.515880	31.249474
	SO + CO-6	64,583	54,877	179	179.17	3.102885	31.079983
SO + GD	SO + GD-1	63,538	50,750	181	181	3.544016	29.533213
	SO + GD-2	54,975	46,775	164	164	3.259715	29.025979
	SO + GD-3	68,486	57,413	201	201.75	3.615350	31.816272
	SO + GD-4	66,992	55,196	212	212.60	3.746470	31.964809
	SO + GD-5	61,770	52,328	138	138	2.567783	27.435939
	SO + GD-6	62,700	52,130	161	161.25	3.000332	27.213688
2K1C + CO	2K + CO-1	68,480	59,072	180	182	3.230878	30.647059
	2K + CO-2	62,608	52,757	171	171	3.227207	29.824251
	2K + CO-3	66,130	53,720	181	181	3.510320	30.163243
	2K + CO-4	62,685	52,017	152	152	3.127591	27.372778
	2K + CO-5	58,580	49,144	168	168.50	3.362523	29.371917
	2K + CO-6	67,403	56,003	153	153.60	2.997668	28.221158
2K1C + GD	2K + GD-1	51,957	44,729	134	134	3.108962	23.048880
	2K + GD-2	76,915	64,610	184	184	2.956281	30.266004
	2K + GD-3	62,825	52,113	184	187	3.888140	30.204312
	2K + GD-4	73,201	62,088	212	212	3.618511	34.156697
	2K + GD-5	75,393	61,743	206	206.86	3.704636	33.153967
	2K + GD-6	74,875	63,476	198	201.33	3.439787	32.834124

## Data Availability

The datasets generated for this study can be found in the NCBI SRA data with accession number of PRJNA895211. Data deposited to MetaboLights with ID-MTBLS6428 (https://www.ebi.ac.uk/metabolights/MTBLS6428).

## Abbreviations

2K1C	2-kidneys-1 clip
ASVs	Amplicon sequence variants
BW	Body weight
CO	Corn oil
GD	*Garcinia dulcis*
LEfSe	Linear discriminant analysis effect size
NMDS	Non-metric multidimensional scaling
PCoA	Principal coordinate analysis
PERMANOVA	Permutational multivariate analysis of variance
PD	Phylogenetic diversity
RVH	Renovascular hypertension
SBP	Systolic blood pressure
SCFA	Short chain fatty acid
SO	Sham operation
UHPLC	Ultra-high performance liquid chromatography
